# Hahnemann’s Influence upon Surgery

**Published:** 1896-09

**Authors:** Howard Crutcher

**Affiliations:** Chicago


### HAHNEMANN'S INFLUENCE UPON SURGERY.

HOWARD CRUTCHER, M. D., CHICAGO.

Samuel Hahnemann said very little about the practice of surgery, but what he did say is of the first importance as indicating the soundness of his reasoning and the value of his teachings.

In an original volume of *The Organon*, published in 1833

(*The Homœopathic Medical Doctrine, or Organon of the Healing Art, A New System of Physic, etc.*, Dublin, W. F. Wakeman, 1833), I find the following in paragraph 183:

"* * * The treatment of these maladies belongs to surgery. So far as it is necessary to bring mechanical aid to the suffering parts in order to remove and annihilate mechanical obstacles to the cure, which can only be expected from the powers of the organism itself. Among these may be ranked, for example, the reduction of dislocations, uniting wounds by bandages, extracting foreign substances that have penetrated the lining parts, *opening the cavity of the abdomen either to remove a substance that is burdensome to the system, or to give vent to effusions and collections of liquids*, placing in apposition the extremities of a fractured bone, and consolidation of the fracture by means of an appropriate bandage, etc."

I do not know in what year these words of Hahnemann were written; but it should be a matter of profound pride that as early as 1833 he advocated an operation which was then almost unknown, and which was opposed at that day by a great majority of old-school surgeons. The first deliberate abdominal operation in the world was performed by Ephraim McDowell, at Danville, Ky., in December, 1809; and it speaks volumes for Hahnemann's far-seeing sagacity that he is found advocating the operation twenty-four years later. It must be remembered that abdominal surgery was in its infancy many years after Hahnemann was in his grave. Instead of opposing the march of surgical practice, Samuel Hahnemann was one of the first to recognize its value and to advocate its adoption. It is a matter of profound regret that many of his professed disciples have not possessed the wisdom to follow in the footsteps of the great teacher.

Reasoning by analogy, it seems perfectly fair to conclude that if it be Hahnemannian practice to drain a peritoneal cavity, it is also legitimate to drain other cavities; that if it be proper to drain off serum, it is still more proper to evacuate pus. Why should a dropsical effusion be removed from the peritoneum and a collection of foul matter be allowed to remain in the triangles of the neck? For my part, I evacuate pus wherever it can be located, whether beneath the periosteum, or in the chest, or in the abdomen, or in the axilla. The same rule applies to certain unsightly tumors, which are "burdensome" to the eye, if not to the system. Yet there are honest men and women who stand aghast at the opening of a felon, at the tapping of a hydrocele, and at the shelling out of a fatty tumor. It is to be deeply regretted that Hahnemann is so often quoted as being opposed to these procedures. In this connection I recall the fact that I was once roundly lectured because I wanted to open the bladder for exploratory purposes where remedies had been tried in vain for months.

There is reckless surgery, as all of us know, and against this kind of practice our voices should be raised in season and out of season; but to suppose that Hahnemann, who was one of the greatest thinkers who ever lived, arrayed himself against the legitimate use of the knife, is to defy the teachings of all human experience. The Hahnemannian surgeon, who is able to look beyond tissues to forces, is thereby enabled to steer clear of many of the pitfalls that lie in the path of his less fortunate colleagues. The medical history of many a patient will reveal the fact that he is not an ideal subject for a surgical operation. There is something more in the problem than dividing tissues, ligating vessels, and running the chance of bacterial infection.

Hahnemann taught clearly the necessity of legitimate mechanical work; he also exposed the absurdity of regarding a carcinoma as a local growth, and one day the world will come to view the pathology of morbid growths in the true light of the Hahnemannian philosophy. We have a great educational work to do along these lines. The pathology of malignant tumors is not understood by old school authorities, and with their present light they never can understand it. But the truth will stand revealed at last. And when the truth shall stand forth in its might and power, great will be the glory of Hahnemann—the pathologist, the healer, the surgeon.

